# Advancing stroke recovery: unlocking the potential of cellular dynamics in stroke recovery

**DOI:** 10.1038/s41420-024-02049-5

**Published:** 2024-07-11

**Authors:** Keivan Sahebi, Hassan Foroozand, Mobina Amirsoleymani, Saghi Eslamzadeh, Manica Negahdaripour, Amir Tajbakhsh, Abbas Rahimi Jaberi, Amir Savardashtaki

**Affiliations:** 1 Student Research Committee, Shiraz University of Medical Sciences, Shiraz, Iran; 2 Department of Pharmaceutical Biotechnology, School of Pharmacy, Shiraz University of Medical Sciences, Shiraz, Iran; 3 Pharmaceutical Sciences Research Center, Shiraz University of Medical Sciences, Shiraz, Iran; 4 Clinical Neurology Research Center, Shiraz University of Medical Sciences, Shiraz, Iran; 5 Department of Neuroscience, School of Advanced Medical Sciences and Technologies, Shiraz University of Medical Sciences, Shiraz, Iran; 6 Department of Medical Biotechnology, School of Advanced Medical Sciences and Technologies, Shiraz University of Medical Sciences, Shiraz, Iran; 7 Infertility Research Center, Shiraz University of Medical Sciences, Shiraz, Iran

**Keywords:** Cellular neuroscience, Stroke, Immune cell death

## Abstract

Stroke stands as a predominant cause of mortality and morbidity worldwide, and there is a pressing need for effective therapies to improve outcomes and enhance the quality of life for stroke survivors. In this line, effective efferocytosis, the clearance of apoptotic cells, plays a crucial role in neuroprotection and immunoregulation. This process involves specialized phagocytes known as “professional phagocytes” and consists of four steps: “Find-Me,” “Eat-Me,” engulfment/digestion, and anti-inflammatory responses. Impaired efferocytosis can lead to secondary necrosis and inflammation, resulting in adverse outcomes following brain pathologies. Enhancing efferocytosis presents a potential avenue for improving post-stroke recovery. Several therapeutic targets have been identified, including osteopontin, cysteinyl leukotriene 2 receptor, the µ opioid receptor antagonist β-funaltrexamine, and PPARγ and RXR agonists. Ferroptosis, defined as iron-dependent cell death, is now emerging as a novel target to attenuate post-stroke tissue damage and neuronal loss. Additionally, several biomarkers, most importantly CD163, may serve as potential biomarkers and therapeutic targets for acute ischemic stroke, aiding in stroke diagnosis and prognosis. Non-pharmacological approaches involve physical rehabilitation, hypoxia, and hypothermia. Mitochondrial dysfunction is now recognized as a major contributor to the poor outcomes of brain stroke, and medications targeting mitochondria may exhibit beneficial effects. These strategies aim to polarize efferocytes toward an anti-inflammatory phenotype, limit the ingestion of distressed but viable neurons, and stimulate efferocytosis in the late phase of stroke to enhance post-stroke recovery. These findings highlight promising directions for future research and development of effective stroke recovery therapies.

## FACTS

- Efferocytosis plays a critical role in maintaining tissue homeostasis and resolving inflammation and has a critical role post-stroke recovery.
- Enhancing efferocytosis has emerged as a promising therapeutic strategy for post-stroke treatment, as it can reduce inflammation, promote tissue repair, and improve neurological outcomes.
- Several potential pharmacological therapeutic targets and approaches for efferocytosis in post-stroke treatment have been identified, including osteopontin, thiazolidinediones, metformin, fluoxetine, cysteinyl leukotriene 2 receptor antagonist, NFκB signaling inhibitor, β-funaltrexamine, annexin A1, mitochondrial targets (e.g., humanin), CDP-choline, sCD163, and microglia-bound CD163.
- Among non-pharmacological approaches, physical rehabilitation, hypothermia, and intermittent hypoxia have shown beneficial effects in post-stroke recovery.
- The potential for personalized approaches in efferocytosis-targeting therapies, considering individual patient factors that may influence the optimal treatment strategy, should be explored to enhance the clinical relevance and applicability of these interventions.

## OPEN QUESTIONS

- What are the key molecular and cellular mechanisms underlying impaired efferocytosis in the context of stroke pathogenesis, and how can these be therapeutically targeted to improve post-stroke recovery?
- How can the various therapeutic targets identified for enhancing efferocytosis be optimized and combined for enhanced efficacy in stroke treatment?
- What is the role of ferroptosis in post-stroke tissue damage and neuronal loss, and how can inhibiting ferroptosis be integrated with efferocytosis-targeting strategies to develop effective combination therapies for improved neurological outcomes?
- Can CD163 and other proposed biomarkers be reliably utilized for early diagnosis, prognostic assessment, and therapeutic monitoring in acute ischemic stroke, and how can these biomarkers guide personalized treatment approaches?
- What are the underlying mechanisms and optimal parameters for non-pharmacological approaches, such as physical rehabilitation, hypoxia, and hypothermia, in modulating efferocytosis and other cellular processes to enhance post-stroke recovery?
- How can therapies targeting mitochondrial dysfunction be leveraged in conjunction with efferocytosis-enhancing strategies to address the multifaceted pathophysiology of stroke and improve overall patient outcomes?

## Introduction

Stroke is a leading cause of death and disability worldwide, arising from disturbed blood flow to an area of the central nervous system (CNS). Brain strokes are primarily classified into two types, including ischemic and hemorrhagic; the former consists of the majority of cases (70–80%) [1]. The current standard treatment option for acute ischemic stroke is the administration of thrombolytic agents [2]. Nonetheless, patients have limited eligibility due to a narrow treatment window [3], and the treatment may be associated with many adverse effects, most importantly hemorrhagic transformation [4]. In certain clinical circumstances, endovascular thrombectomy may be considered, but it carries significant risks of complications and mortality [5]. Patients experiencing brain stroke have variable degrees of neuronal loss and suffer from a wide range of lifelong complications and functional disabilities [6–8]. At present, among non-pharmacological options, physical rehabilitation has shown effectiveness in improving functional recovery after stroke [8]. However, the exact underlying mechanism and molecular basis of non-pharmacological strategies are not clear.

**Fig. 1:**
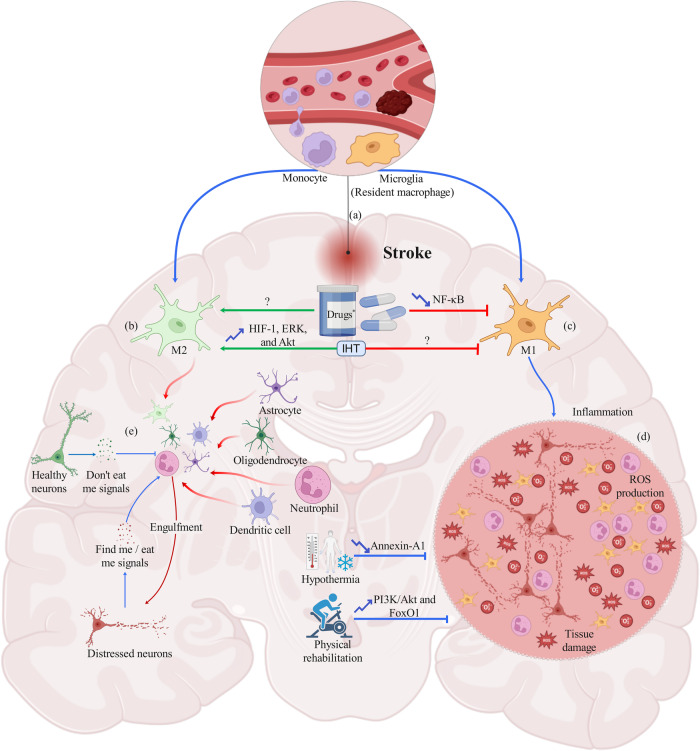
A schematic illustration of post-stroke efferocytosis. After the occurrence of a brain stroke, local microglia (resident macrophages) and blood-derived monocytes enter the insulted area and undergo various genetic and molecular differentiations, resulting in two main phenotypes of activated macrophages: M1 and M2 (**a**). The M2 phenotype exerts anti-inflammatory activity (**b**), while the M1 phenotype is primarily a pro-inflammatory phagocyte (**c**), initiating extensive inflammation, ROS production, and tissue damage (**d**). The activated M2 microglia and other activated efferocytes, such as astrocytes, dendritic cells, neutrophils, and oligodendrocytes, irreversibly eliminate distressed neurons to avoid escalation of inflammation and tissue damage (**e**). Effective efferocytosis protects bystander healthy neurons from being phagocytosed and may even rescue distressed but viable neurons *via* cytokine production (e.g., IL-10 and -4). * Recent studies have investigated the efficacy of some medications on M2/M1 polarization, such as OPN, glucose-lowering agents (metformin, TZDs), CysLT2R antagonists, NF-κB inhibitors, β-funaltrexamine, miR-98, PPARγ, RXR agonists, CDP-choline, and IL-4. Although their exact mechanism of action is still unclear, the majority of these medications may have some inhibitory effects on the NF-κB signaling pathway. Among non-pharmacological treatments, IHT may increase HIF-1, ERK, and Akt signaling pathways and reduce M1 polarization, ROS production, and tissue damage. Physical rehabilitation, especially if not initiated early after the occurrence of stroke, may have inhibitory effects on inflammation, probably through the PI3K/Akt and FoxO1 signaling pathways. Post-stroke hypothermia has various anti-inflammatory effects; one important target might be Annexin A1 in PMN cells. ROS reactive oxygen species, PMN polymorphonuclear cells, OPN osteopontin, TZDs thiazolidinediones, CysLT2R cysteinyl leukotriene receptor 2, NF-κB nuclear factor kappa-light-chain-enhancer of activated B cells, HIF-1 hypoxia-inducible factor 1, ERK extracellular signal-regulated kinase, Akt protein kinase B, PI3K phosphoinositide 3-kinase, FoxO1 forkhead box protein O1, miR-98 microRNA-98, PPARγ peroxisome proliferator-activated receptor gamma, RXR retinoid X receptor, CDP-choline cytidine-5V-diphosphocholine, IL interleukin.

**Fig. 2:**
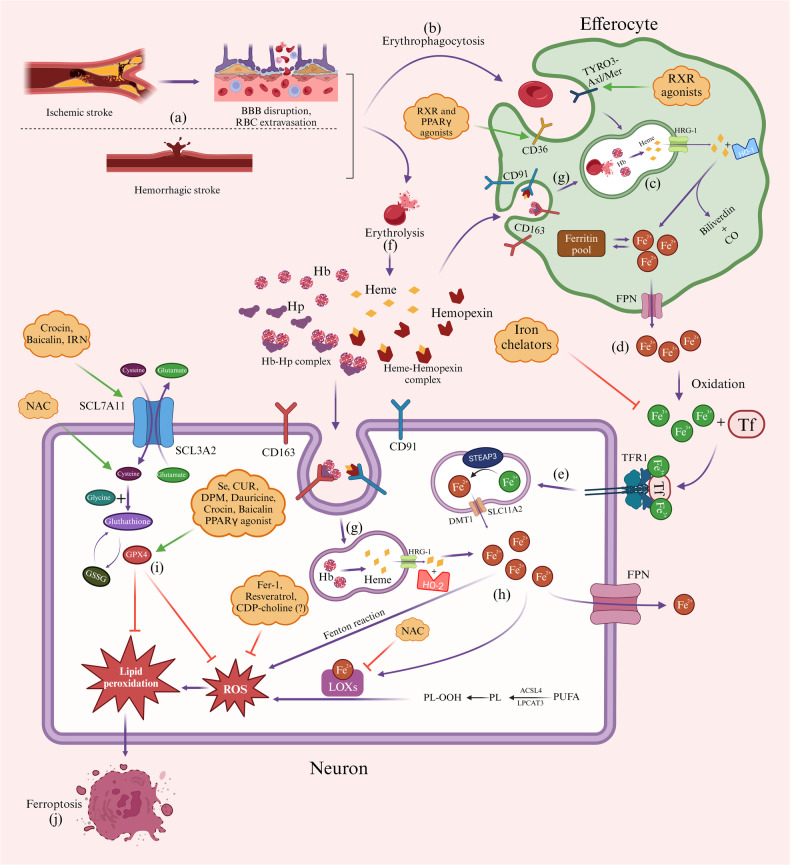
A schematic illustration of post-stroke ferroptosis and related therapeutic targets. After a hemorrhagic stroke, numerous RBCs are released in the affected region. Moreover, BBB integrity is disrupted following an ischemic stroke, leading to extravasation of RBCs (**a**). These RBCs are either phagocytosed by local immune cells (**b**) or undergo lysis (**f**). CD36 and Axl are two important regulators of erythrophagocytosis, upregulated by RXR and PPARγ agonists (**b**). The phagocytosed RBCs are digested in the phagolysosome, and the resultant heme is exported via HRG-1 (**c**). Heme is then hydrolyzed into Fe^2+^, CO, and biliverdin by HO-1. Fe^2+^ ions are then incorporated into ferritin or transported into extracellular space through FPN (**d**). Fe^2+^ is then oxidized into Fe^3+^ and forms Tf-Fe^3+^ complexes, which are uptaken by local neurons (**e**). Iron chelators reduce iron load (**d**) and diminish its neurotoxic effects. In addition, lysed RBCs (**f**) release massive amounts of Heme and Hb, which are uptaken by both efferocytes and neurons via CD91 and CD163, respectively (**g**). The Fe^2+^ load in the neurons accelerates ROS production and lipid peroxidation through several pathways (**h**). A protective pathway, cysteine-gluthatione-GPX4, scavenges the ROS and ameliorates lipid peroxidation (**i**). The path is the target of many medications, enhancing intracellular anti-oxidant activity. Finally, the massive production of ROS and lipid peroxidation leads to cell death and completes the ferroptosis process (**j**). BBB blood-brain barrier, RBC red blood cell, RXR retinoid X receptor, PPARγ peroxisome proliferator-activated receptor gamma, Hb hemoglobin, HRG-1 heme-responsive gene 1, CO carbon monoxide, FPN ferroportin, HO-1 and -2 heme oxygenase-1 and -2, Tf transferrin, TFR1 transferrin receptor 1, STEAP3, the six-transmembrane epithelial antigen of prostate family member 3, DMT1 divalent metal transporter 1, NAC N-acetylcysteine, IRN isorhynchophylline, Fer-1 ferrostatin-1, CDP-choline cytidine-5V-diphosphocholine, LOXs lipoxygenase, PUFA poly-unsaturated fatty acid, PL phospholipid, Se selenium, CUR curcumin, GPX4 glutathione peroxidase 4, ROS reactive oxygen species, SLC7A11 solute carrier family 7 member 11, SLC3A2 solute carrier family 3 member 2, SLC11A2 solute carrier family 11 member 2.

Stroke is a complex series of genetic, molecular, and cellular processes [9] (Fig. 1). Immediately after the occurrence of ischemia, whether due to thromboembolic or hemorrhagic causes, polymorphonuclear (PMN) cells and monocytes enter the ischemic area and attempt to restrict the tissue damage and clear the distressed cells [10]. A particular focus is now placed on the anti-inflammatory phenotype of macrophages, known as the M2 phenotype, and the role of effective efferocytosis, which is the hemostasis process by which the immune system removes distressed and apoptotic cells (ACs) to reduce the escalation of inflammation and lessen neuronal loss [11, 12] (Fig. 1). Moreover, ferroptosis is an emerging target for molecular treatments to attenuate neuronal loss following iron toxicity (Fig. 2). Recently, various studies have attempted to discover therapeutic molecular targets to enhance M2 polarization, reduce infarct volume, and improve functional outcomes [13–15]. In addition to physical rehabilitation [16] and signaling pathways associated with the phagocytic activity of microglia and macrophages, recent studies have addressed important therapeutic roles for intermittent hypoxia training (IHT) [17] and hypothermia [18]. Mitochondria, as the source of reactive oxygen species (ROS) and initiator of cell apoptosis, has a central role in the molecular and clinical course of stroke [19, 20].

In this review, we will discuss (i) current knowledge on the molecular basis of efferocytosis as the cornerstone target of novel therapies for stroke and its future directions; (ii) current evidence on the function of CD163 as a game-changing target and a novel biomarker of acute stroke; (iii) molecular pathways and potential targets involved in physical rehabilitation, hypothermia, and IHT after stroke; and (iv) the emerging role of mitochondrial dysfunction and mitochondria-targeted therapies.

## Post-stroke efferocytosis

### Efferocytosis as a brain homeostatic process

Efferocytosis is a brain homeostatic mechanism responsible for the removal of ACs that have accumulated within the brain tissue due to diverse pathological conditions [11]. This process possesses neuroprotective and immunoregulatory effects in the insulted area of the brain. The phagocytes driving efferocytosis consist of cells with a primary efferocytosis function, known as “professional phagocytes,” including microglia, macrophages, neutrophils, and dendritic cells [21], or those capable of efferocytosis under certain conditions, such as oligodendrocytes, referred to as “non-professional phagocytes” [22, 23]. The quick and timely removal of ACs prevents their death through subsequent necrotic processes. When necrosis is initiated, intracellular components are released into the surrounding tissue, thereby inciting an inflammatory response and local pathologies [24].

In addition to suppression of pro-inflammatory and induction of anti-inflammatory mediators [25–27], phagocytosis of ACs enhances the production of factors involved in the resolution of inflammation [28] and tissue repair, such as bioactive lipids [29] and growth factors [25, 30].

Efferocytosis occurs through four major steps: “Find-Me,” “Eat-Me,” “engulfment/digestion,” and “anti-inflammatory responses.” “Find-Me” signals, varying according to the type of injury and the tissue [31], are released by dead cells. These signals include sphingosine-1-phosphate (S1P), lysophosphatidylcholine (LPC), nucleotides adenosine triphosphate (ATP), CX3C motif chemokine ligand 1 (CX3CL1), and uridine triphosphate (UTP) [32–35]. Distressed cells produce “Eat-Me” signals, such as phosphatidylserine (PtdSer), intracellular adhesion molecule 3 (ICAM-3), and calreticulin (Calr) [36–39]. Meanwhile, healthy cells express “Don’t Eat-Me” signals by producing CD31, SIRPα, and sialic acid-binding Ig-like lectin 10 (Siglec-10) to avoid phagocytosis [39, 40]. Phagocytes have specific surface receptors for these signals, and several soluble proteins, including milk fat globule EGF factor 8 (MFG-E8), growth-arrest specific 6 (Gas6), protein S (ProS), and C1q, provide the linkage between the phagocyte and ACs [39]. Next, the identified ACs should be engulfed by phagocytes. Two important regulators of this phase are Rho-family GTPases Rac Family Small GTPase 1 (Rac1) and Ras Homolog Family Member A (RhoA). Rac1 initiates actin reorganization and promotes phagocytosis, while RhoA exerts inhibitory effects [41]. Subsequently, the phagosome proceeds through additional maturation steps, leading to the activation of various signaling pathways responsible for the breakdown of the internal contents [42]. However, different profiles of signaling proteins and phagocytic receptors are expressed in different tissues and ACs [43, 44], resulting in tissue- and AC-specific efferocytosis.

In the hypoxic-ischemic brain, a key pathway of efferocytosis signals is the interleukin-4 (IL-4)-signal transducer and activator of transcription 6 (STAT6)- peroxisome proliferator-activated receptor-gamma (PPARγ)-arginase-1 (Arg-1) (IL-4-STAT6-PPARγ-Arg1) signaling axis [45]. Resident microglia are the major efferocytes of the brain, both in normal and pathologic conditions [46]. They are the first phagocytes to reach the injury site and form a protective barrier. The ischemia triggers genetic reprogramming in efferocytes [47], resulting in their proliferation, polarization, and phagocytic enhancement through the upregulation of surface receptors, extracellular bridge molecules, cytoskeletal rearrangements, and the synthesis of anti-inflammatory and trophic factors [47, 48].

### Efferocytosis is a timely cascade of events

Within the first 24 hours of ischemia, microglia enter the core of the injury site and start the efferocytosis process [49] by phagocytosing dendrites, cell bodies, and nuclei. After about three days post-stroke, the penumbral is occupied by activated astrocytes that engulf synaptic debris [50, 51]. A tightly regulated crosstalk between microglia and astrocytes derives the effective clearance of injured neurons [52–55]. Astrocytes are capable of impeding the efferocytic capability of microglia [56] or promoting their shift toward an anti-inflammatory phenotype through the release of exosomes carrying miR873a-5p [57]. Microglial P2Y1 receptor downregulation induces a shift in astrocytes toward a neuroprotective phenotype [58]. Another important communication pathway is the microglia-pericyte crosstalk. Pericytes attract macrophages and boost their phagocytic capabilities. In contrast, macrophages secrete trophic factors and amplify platelet-derived growth factor receptor β (PDGFRβ) signaling in pericytes, promoting the synthesis of extracellular matrix proteins [59]. After AC removal, oligodendrocyte precursor cells, or neuron-glial antigen 2-expressing glia (NG2 glia), occupy the vacated niche, supporting neuroregeneration through processes such as remyelination and potential transformation into neurons or astrocytes [60–62].

After brain ischemia, microglia undergo differentiating into either the M1 or M2 phenotype. M1 microglia are associated with pro-inflammatory functions, while the M2 phenotype is characterized by its anti-inflammatory and protective functions [63, 64]. The M2 phenotype signifies a comparatively favorable behavior that facilitates the resolution of inflammation through the clearance of cellular remnants and harmful substances, thereby promoting tissue repair and facilitating recovery after a stroke. It’s important to keep in mind that the M1/M2 classification is a simplification of a more complex spectrum. The polarization is highly affected by the signals and tissues, resulting in a range of immunophenotypes with overlapping properties. More importantly, multiple subsets of M2 macrophages (e.g., M2a, M2b, M2c, and M2d) and hydride phenotypes have been recently addressed [65]. However, many aspects of these intermediate phenotypes are still unknown, and the M1/M2 concept presented here and in other research articles is used to imply the overall picture.

Defective efferocytosis, either excessive or insufficient, causes necrosis and inflammation and may be responsible for adverse outcomes after brain pathologies. Thus, treatments aiming at polarizing efferocytes to the anti-inflammatory phenotype, limiting the ingestion of distressed yet viable neurons in the initial phase of ischemia [66], and stimulating efferocytosis in the late phase of stroke could enhance post-stroke recovery [67]. Here, we will discuss recent therapies potentiating post-stroke efferocytosis. We will also focus on two emerging diagnostic and prognostic targets: the underlying risk factors and novel biomarkers.

### Potential efferocytosis-directed therapeutic targets

In recent studies, many pharmacological treatments have been introduced as effective treatments to enhance post-stroke recovery, either alone or as adjunctive therapies. Osteopontin (OPN) has shown promising effects on M2 microglia polarization following cerebral ischemia. It enhanced the effective efferocytosis of ACs in a rat middle cerebral artery occlusion (MCAO) model [68, 69]. Through the Fak, ERK, and Akt signaling pathways, an OPN derivative peptide, known as arginine-glycine aspartic acid (RGD)-containing 7-amino-acid OPN peptide (OPNpt7R, or VPNGRGD), was shown to enhance the efferocytic activity and motility of microglia [70]. In addition, intranasal RGD-containing Osteopontin Heptamer Peptide was able to reduce infarct volume and improve neurological function in a rat model of MCAO. The peptide polarized microglia toward the M2 phenotype, as evidenced by the suppression of M1 markers and upregulation of M2 markers in the immunohistochemistry (IHC) study [71].

Recently, some glucose-lowering agents have been introduced as effective treatments for M2 polarization, possibly through mechanisms independent of their glucose-lowering activities. For example, rosiglitazone and pioglitazone, two thiazolidinediones (TZD) drugs, are able to induce phenotype switching in microglia via suppressing mitogen-activated protein kinase (MAPKs) and nuclear factor kappa-light-chain-enhancer of activated B cells (NF-κB) [72], leading to a significant reduction in infarct volume and improved neurological function [73, 74]. Similar beneficial outcomes were achieved by administering two doses of troglitazone or pioglitazone 24 h before and again at the time of MCAO in rat models [75]. The study also showed that infarct volume and neurological assessment continued to improve in the pioglitazone group over a period of 22-day follow-up.

In addition to TZDs, microglia activation and M2 polarization following brain stroke could be induced by metformin, a glucose-lowering agent belonging to biguanides [76].

Two novel therapeutic agents, cysteinyl leukotriene 2 receptor (CysLT2R) selective antagonist HAMI3379 and NF-κB signaling inhibitor Bay 11-7082, were capable of M1 suppression and induction of M2 polarization [77]. The CysLT2R and NF-κB pathways are responsible for M1 polarization after cerebral ischemia (Fig. 1), and inhibition of these signaling pathways may exert beneficial effects. In addition, β-funaltrexamine, a µ opioid receptor antagonist, has shown anti-inflammatory and neuroprotective effects in a rat model of cerebral ischemia/reperfusion (I/R) injury. The underlying molecular mechanism is probably through suppressing NF-κB, activator protein 1 (AP-1), and cyclic adenosine monophosphate (cAMP) response element-binding protein (CREB). β-funaltrexamine increased the M2/M1 ratio and decreased neuroinflammation and neuronal loss through suppressing tumor necrosis factor-α (TNF-α), IL-1 β, nitric oxide (NO), and prostaglandin E2 (PGE2), along with upregulation of Arg-1 and CD163 (M2) [78].

Some other medications with anti-inflammatory properties may include phthalide, CD21, and IL-4. The anti-inflammatory and neuroprotective activities of phthalide after brain ischemia are probably associated with the inhibition of damage-associated molecular pattern (DAMP)/Toll-like receptor 4 (TLR4) pathway [79]. Treatment with CD21 was shown to reduce infarct volume and enhance neurological outcomes in an MCAO rat model. It has been suggested that the neuroprotective effects of CD21 are mediated through inhibition of the TLR4/NF-κB pathway and induction of macrophage scavenger receptor 1 (MSR1)-promoted DAMP (PRX1) clearance [80]. IL-4 is released from distressed neurons and enhances the efferocytic activity of microglial cells. IL-4 administration has been shown to decrease infarct volume and improve post-stroke outcomes [81].

Although studies on microRNAs (miRNAs) promoting M2 polarization and efferocytosis are few, extracellular vesicles loaded with miR-98 could rescue the stressed but viable neurons from phagocytosis. It has been claimed that miR-98 mediates efferocytosis and exerts its neuroprotective activity through the platelet-activating factor receptor (PAFR) [82]. Fluoxetine is a known antidepressant medication with immunomodulatory effects. It has been reported that fluoxetine decreases the production of pro-inflammatory cytokines, attenuates microglial oxidative stress, and facilitates efferocytosis [83].

### Post-stroke efferocytic responses are influenced by underlying risk factors

Several risk factors have been shown to not only increase the incidence of stroke, but also worsen its outcome and mortality. Recent molecular studies have suggested that a “primed” inflammatory environment may exist in the brains of individuals with stroke risk factors [84]. These risk factors mostly belong to chronic vascular and metabolic syndromes, namely obesity, arterial hypertension, atherosclerosis, and diabetes. For instance, compared to the control lean strain, obesity and atherosclerosis in rats led to microgliosis and increased microglia activation on positron emission tomography (PET) imaging [84]. In addition, in *ApoE*^*-/-*^ mice fed with an atherogenic diet, the expression of vascular adhesion molecules, such as ICAM and vascular intracellular adhesion molecule (VCAM), was increased. These adhesion molecules were also positively correlated with higher infiltration of CD45^+^ leukocytes (most commonly granulocytes) in the choroid plexus.

Regarding post-stroke outcomes, hypertensive rats showed significantly larger infarct volumes and higher brain leukocyte infiltration compared to normotensive animals [85]. However, the M1/M2 polarization was not affected by hypertension. The study found a similar proportion of CD80+ (M1) and CD163^+^ (M2) macrophages as well as typical M1 markers (e.g., IL-1β, IL-6, and TNF-α) and M2 markers [e.g., IL-10, matrix metallopeptidase (MMP)-9, and transforming growth factor-β (TGF-β)] between hypertensive and normotensive rats. Exploring the association between obesity and cerebral inflammation after stroke, obese mice fed a high-fat (60%) diet showed larger post-stroke infarct volumes and increased chemokine expression (CXCL-1 and CCL3), blood-brain barrier (BBB) permeability, and neutrophil and microglia counts [86].

Of note, gender is an important factor influencing different immune responses following stroke. Production of pro-inflammatory cytokines (TNF-α, IL-1β, and CCL3) from post-MCAO microglia in female mice was reduced more dramatically in response to recombinant IL-10 and IL-10^+^ B-cells. Moreover, IL-4R expression and IL-4 production in female-derived microglia were significantly higher than in microglia from male animals. Induction of M2 (CD206^+^) polarization was more efficient in female microglia treated with recombinant IL-4 [87], indicating greater sensitivity of female microglia to this cytokine.

### Post-stroke biomarkers of efferocytosis: an opportunity to individualize the treatment

In addition to novel therapeutic targets, recent studies have introduced numerous diagnostic and prognostic biomarkers for stroke [88]. Several biomarkers, such as IL-6 [89], TNF-α, ICAM-1, MMP-2, MMP-13 [90, 91], and complement C3 [92], have been identified as being associated with poor outcomes or larger infarct volumes. As therapeutic guides, higher levels of soluble endothelial protein C receptor and soluble thrombomodulin are reported to be associated with recanalization failure following tPA treatment [93]. These biomarkers are originally endothelial receptors involved in inflammation and the coagulation process. Similarly, elevated MMP-9 levels could serve as predictors of thrombolysis failure [94]. Along with fibronectin, MMP-9 is a good predictor of vascular damage and hemorrhagic transformation following tPA administration [95].

However, the prognostic and therapeutic aspects of a few efferocytosis-associated biomarkers have been investigated [96]. In the following, we will discuss in more detail the three receptors/biomarkers associated with efferocytosis, including CD36, CD91, and CD163. They are mostly involved in post-stroke erythro-/heme-/Hb-phagocytosis. Here, we focus on their soluble form (*i.e*., sCD91, sCD36, and sCD163) as potential biomarkers to improve patient outcome or therapeutic goals, although studies on CD63 and CD91 are scarce.

#### CD36

The cluster of the differentiation 36 receptor (CD36) is a transmembrane glycoprotein receptor with multifunctional properties [97–100]. The receptor is a crucial part of erythrophagocytosis and hematoma clearance [97]. After a hemorrhagic stroke, CD36 has been reported to be associated with hematoma absorption, less neuronal injury, and better clinical outcomes [97, 101], making its soluble form (sCD36) an appropriate biomarker candidate for risk stratification in ICH patients. Apart from phagocytosis of extravasated RBCs, it is suggested that CD36 may participate in scavenging several lipoproteins, including high-, low-, and very low-density lipoproteins (HDL, LDL, and VLDL), oxidized LDL, long-chain fatty acids (LCFA), or advanced glycation end products (AGEs) [98–100]. It is possible that, contrary to ICH, elevated CD36/sCD36 are associated with enhancement of foam cell formation, leading to progression of inflammation, atherosclerosis [102], plaque destabilization, thrombus formation, and higher incidents of ischemic events [103]. In addition, with similar reasoning, sCD36 may be associated with vascular damage and exacerbated outcomes of ischemic stroke.

#### CD91

The cluster of the differentiation 91 receptor (CD91), also known as low density lipoprotein receptor-related protein 1 (LRP1) receptor, is another multifunctional transmembrane receptor for endocytosis of the heme-hemopexin (Hb-Hp) complex [104]. Thus, cell-surface CD91 may be involved in heme scavenging following an ICH event and diminishing inflammatory and ferroptotic sequelae. Contrary to the anti-ferroptotic properties of membrane-bound CD91, the soluble form (sCD91) participates in microglia activation and induction of a pro-inflammatory state [105]. Moreover, CD91 may act with Mac-1 to activate latent platelet-derived growth factor-CC (PDGF-CC) and the PDGF receptor-alpha (PDGFRα), leading to accelerated tPA-induced BBB dysfunction and microglia activation [106]. Together, it seems that CD91 and sCD91 are two levers of neuroinflammation and have both pro- and anti-inflammatory properties. The actual utility of this biomarker for risk stratification and therapeutic targets should be evaluated by future studies.

#### CD163

The CD163 receptor is a 130-kDa membrane protein expressed on the cell surfaces of monocytes and macrophages [107, 108]. CD163 is highly expressed both on M1 and M2 macrophages, which are responsible for inflammatory and counter-regulatory responses, respectively. While M1 macrophages are induced by cytokines such as IFNγ, IL-12, and IL-18 released from Th1 lymphocytes and natural killer (NK) cells, IL-4 and IL-13 are primarily responsible for the induction of M2 macrophages [107]. A distinct phenotype originated from M1 macrophages, known as Mhem, with high levels of iron and heme oxidase 1 (HO1) has been introduced. In these macrophages, CD163 acts as a hemoglobin scavenger receptor. CD163 binds to the hemoglobin-haptoglobin complex with high affinity, while its affinity for free hemoglobin is low. After binding, the complex is endocytosed and degraded to heme metabolites, such as bilirubin, Fe^2+^, and carbon monoxide (CO) [109, 110]. The CD163 receptor is also present in neurons. After a hemorrhagic or ischemic stroke, neuronal C163 could uptake the released hemoglobin, leading to neurotoxicity and neuronal loss [111]. As a result, soluble CD163 (sCD163) and microglia-bound CD163 could, theoretically, sequestrate hemoglobin and reduce the inflammatory and toxic effects of hemoglobin and iron on neurons [112–114].

Immunohistologic studies on atherosclerotic plaques have shown that symptomatic plaques are rich in Th1 cytokines and M1 macrophages, while Th2 cytokines, M2 macrophages, and CD163 are expressed more in stable plaques [115, 116]. Paradoxically, in another study, CD163 was significantly upregulated in symptomatic carotid atherosclerotic plaques and was associated with intraplaque hemorrhage, plaque ulceration, and markers of apoptosis and proliferation, such as activated caspase 3, TUNEL, and Ki67 [117]. CD163 expression might be affected by a stroke. In a cross-sectional study by Greco et al., the percentage of peripheral CD163^+^/CD16^+^ monocytes 24 h after the stroke showed a positive association with the severity of stroke at admission [118]. Additionally, another study by Sun et al. [119] reported that patients with acute ischemic stroke had higher levels of serum CD163 than the controls.

CD163 has been introduced alongside nine other genes (*ANTXR2*, *STK3*, *PDK4*, *MAL*, *GRAP*, *ID3*, *CTSZ*, *KIF1B*, and *PLXDC2*) as a specific genetic expression pattern for the diagnosis of acute ischemic stroke [120–122]. This pattern of gene expression in peripheral blood was able to identify acute ischemic stroke cases with good sensitivity (94.7%) and specificity (100%). Moreover, other studies using bioinformatics gene analysis have found CD163 as a potential biomarker [123] for unstable atherosclerotic plaque and ischemic stroke [124]. It is proposed that plasma CD163 may have a prognostic potential since patients with higher plasma CD163 levels had better outcomes [119]. It is noteworthy that ischemia can induce genetic reprogramming in CD163^+^ macrophages, leading to leukocyte chemotaxis and derangements in blood-brain barrier integrity. After transient MCAO, CD163 macrophages upregulated the expression of the hypoxia-inducible factor-1 (HIF-1) pathway, vascular endothelial growth factor (VEGF), inducible nitric oxide synthase (iNOS), and chemoattractants, leading to neurological impairment [125, 126].

## Ferroptosis, a novel therapeutic target

Ferroptosis is defined as an iron-dependent cell death. Iron is an essential element for the proper function of many enzymes. However, it can also catalyze the production of ROS and lead to cell damage [127]. The extravasated hemoglobin after a hemorrhagic stroke is phagocytized by activated efferocytes, degraded to heme, and finally oxidized to Fe^2+^ [128, 129]. The primary enzyme catalyzing heme oxidation to Fe^2+^ is HO. Astrocytes and microglia express HO-1, while HO-2 is mainly expressed in neurons [129, 130]. The function of HO is essential for heme elimination, as it is also a toxic substance to neurons [131]. However, HO is a double-edged sword, and dysregulated HO function may lead to increased Fe^2+^ production that can not be handled by the local reticuloendothelial system, resulting in neurotoxicity.

After a hemorrhagic stroke, the expression of iron-handling proteins, including transferrin, ferritin, and TfR, along with both iron-importing and -exporting proteins, divalent metal transporter 1 (DMT1) and ferroportin (FPN), is increased [132–134]. The oxidized Fe^2+^ is then released out of efferocytes and enters local neurons via the Tf-TfR system. Accumulation of Fe^2+^ results in ROS generation, oxidative stress, damage to nucleic acids, proteins, and lipid membranes, and eventually causes neuronal loss [128].

In light of novel treatments for ICH, a long list of medications aiming at manipulating ferroptosis at multiple molecular levels have shown promising results [135]. Iron chelators reduce the iron load and attenuate its devastating sequelae on neurons. Some well-studied agents may include deferoxamine, pyridoxal isonicotinoyl hydrazone, minocycline, lactoferrin, dexrazoxane, clioquinol, and deferiprone [136–140]. In addition, enhancement of erythrophagocytosis may also lead to better handling of iron and diminishing neurotoxicity. Loss of receptor tyrosine kinases Axl and Mer tyrosine kinase (MerTK), two essential proteins modulating macrophage phenotype differentiation and erythrophagocytosis, led to decreased erythrocyte efferocytosis, increased iron accumulation, and exacerbated neurological dysfunction in a mouse model of ICH [141]. Along with Axl, CD36 is a key receptor regulating erythrophagocytosis. Retinoid x receptor (RXR) agonist bexarotene is able to upregulate both Axl and CD36, leading to improved hematoma absorption and functional recovery [142, 143]. Moreover, the PPARγ agonist, rosiglitazone, exerted similar neuroprotective effects and enhanced hematoma clearance by increasing CD36 expression and effective erythrophagocytosis [144].

Unlike apoptosis, ferroptosis could be reversed by removing its inducers (e.g., by using iron chelators) and adding specific rescuers, such as ferrostatin-1 (Fer-1) or glutathione (GSH) [145]. Fer-1 and resveratrol are two inhibitors of lipid peroxidation, attenuating cellular damage following Fe^2+^ release [146, 147]. In addition, Fer-1 is able to reduce iron accumulation, prevent neuronal loss, and improve neurological outcomes after a hemorrhagic stroke [148, 149].

GSH also inhibits ROS production and prevents various subsequent protein and lipid damage. Studies have shown that stroke may lead to decreased GSH synthesis through inhibition of glutathione peroxidase 4 (GPX4) [150–152]. In a study by Zhang et al. [152] the authors showed that genetic overexpression of GPX4 attenuated oxidative stress and tissue inflammation and improved neuronal function after a hemorrhagic stroke in a rat model. They showed that administration of a GPX4 inhibitor, RAS-selective lethal (RSL)-3, was associated with exacerbated outcomes and brain injury after ICH. In addition, several agents upregulating GPX4, including selenium (Se) [153], dauricine [154, 155], baicalin [156], crocin [157], and curcumin (CUR) [158], have shown beneficial effects after the occurrence of hemorrhagic stroke. Pioglitazone, a PPARγ agonist, probably acts in synergy with the Nrf2/ARE-GPX4 pathway to augment the cellular anti-oxidant systems [159]. Moreover, N-acetylcysteine (NAC) may provide cysteine and help the restoration of GSH resources or inhibit lipoxygenase (LOXs) and lipid peroxidation [160]. Isorhynchophylline (IRN) may exert its neuroprotective activity through activation of the miR-122-5p/TP53/SLC7A11 pathway and increase intracellular cysteine content [161].

A commercially available treatment for stroke is cytidine-5V-diphosphocholine (CDP-choline, citicoline, or Somazina). The medication is now available in some European countries, Spain, and Italy. CDP-choline has shown some beneficial effects in animal and human studies [162–166]; however, the results of controlled clinical trials are still controversial [167–170]. The exact mechanism of CDP-choline neurorecovery is unclear. One possible mechanism is that during cerebral ischemia and hypoxia, neuronal phosphatidylcholine (PC) breaks into choline and free fatty acids (FFA), which in turn are used in ROS production [171–173]. Administration of exogenous choline (as CDP-choline) reverses this pathway, resulting in decreased PC breakdown and FFA oxidation [174, 175]. The key steps in ferroptosis and possible therapeutic targets are summarized in Fig. 2.

## Mitochondria-targeted drugs

Along with efferocytosis and ferroptosis, mitochondrial dysfunction is another source of post-stroke pathologies and poor outcomes. Abnormal oxidative stress and ROS generation may constitute two major components of mitochondrial dysfunction [176]. Although preclinical studies of some mitochondria-targeted agents had serious quality shortcomings [177], others may have promising potential. For instance, stilbazulenyl nitrone (STAZN) is a potent antioxidant and a free radical scavenger. The intravenous injection of STAZN to MCAO rats led to reduced infarct volumes and improved neurological outcomes, providing neuroprotection even at low doses (0.7 mg/kg). Moreover, the agent seems to possess great lipophilicity, making it more effective in penetrating BBB [178]. Administration of resveratrol, a natural compound found in certain foods, beverages, and supplements, has demonstrated neuroprotection against ischemia in rats *via* the SIRT1 uncoupling protein 2 (SIRT1-UCP2) pathway [179]. MRS2365 and 2-methylthioadenosine diphosphate trisodium salt (2meSADP) are substances that can confer neuroprotection after stroke by activating purinergic (P2Y1) receptors on astrocytes, leading to an increase in the metabolic activity of mitochondria within these astrocytes. Administration of 2meSADP caused a significant reduction in the infarct size [180, 181]. Furthermore, administration of 2meSADP decreased edema and increased neuronal survival [182].

Following ischemic stroke, astrocytes release functional mitochondria [183] and a mitochondrial bioactive peptide called humanin [184, 185]. Jung et al. [186] found that ICH is associated with humanin loss in the affected parts of the brain. Injection of intravenous human in mice models of ICH could lead to a reduction of neurological impairments and an improvement in hematoma clearance. The exact mechanisms of functional mitochondria and humanin release by astrocytes are not clear. However, studies suggest that the uptake of these substances by other cells may increase the expression of PPARγ and its related target genes, such as mitochondrial superoxide dismutase. This process can at least increase phagocytosis of red blood cells and lead to reduced iron toxicity and inflammatory responses.

## Non-pharmacological approaches

Apart from pharmacological approaches, non-pharmacological pre- and post-conditioning strategies have shown promising effects on stroke [8]. Physical rehabilitation is now approved as a standard post-stroke therapy, aiding in clinical recovery and neuroplasticity. In addition, hypoxia/ischemia and hypothermia approaches are emerging strategies to improve patients’ prognosis [187, 188]. Although studies are scarce, here we discuss current knowledge on the underlying molecular pathways and provide a helpful review for further research and molecular targeting.

### Physical rehabilitation

As an important treatment strategy, physical rehabilitation has been addressed as a protective approach for stroke outcomes [16]. By activating the forkhead box O (FoxO) and SIRT1/FoxO1 signaling pathways, exercise could reduce cell death and neural loss, increase brain-derived neurotrophic factor (BDNF) production, and promote neuroplasticity and functional recovery after the stroke [189, 190]. Exercise is known to exert its therapeutic effects probably through targeting the FoxO1 protein [191]. It also mitigates ischemia-induced brain damage through upregulating Bcl-2 expression and downregulating caspase-3 and BAX expression [192, 193]. PI3K/Akt signaling, as a mediator of FoxO1, is activated after cerebral ischemia-reperfusion injury [194] and may reduce cerebral injury following focal cerebral ischemia [195–197].

In addition, aerobic exercise pre-conditioning (swimming or treadmill running) 3 weeks before MCAO was able to upregulate BDNF receptor, tropomyosin receptor kinase B (TrkB), as well as TNF-α and MMP-2 [198]. It could be postulated that by increasing controlled inflammatory stress during exercise pre-conditioning, some reparative pathways, such as BNDF, are activated and lead to better outcomes. In another study on a mouse model of ICH, six weeks of treadmill exercise pre-conditioning increased numbers of CD36^+^/Iba-1^+^ microglia (phagocytic microglia), reduced lesion volumes, and promoted post-stroke recovery. Similarly, the pre-conditioning strategy upregulated the plasma levels of some soluble factors, such as endostatin, insulin-like growth factor-binding protein (IGFBP)-2 and -3, MMP-9, OPN, and pentraxin-3 [199].

In animal studies by Feng et al. [200, 201], both mild and intense exercises 24 h post-stroke were associated with a decrease in infarct size, neuron loss, reactive oxygen species (ROS) production, and AC death through SIRT1 and ROS/ER stress pathways. In addition to increased levels of neuroplasticity-associated proteins, post-stroke exercise, irrespective of its intensity, was able to improve the motor and cognitive function of survivor animals. In search of optimal timing for exercise initiation, the authors showed that post-stroke early exercise (6 h) in rats leads to higher oxidative stress through increased ROS and decreased nicotinamide adenine dinucleotide (NADH) and ATP, leading to larger infarct volumes and apoptosis. On the other hand, late exercise initiation (24 h or three days) was associated with smaller infarct sizes and apoptosis, as well as lesser oxidative metabolism [202]. Although it is known that hypermetabolism [203] and accelerated ROS generation [204] are the possible responsible mechanisms by which early exercise causes neuronal loss [205], the beneficial effects of late exercise are probably due to the induction of angiogenesis after the resolution of the acute hypermetabolic environment in early post-stroke.

### Hypoxia/ischemia

Hypoxia can be considered one of the most damaging factors after a stroke. Hypoxia exhibits a devastating effect by triggering proteolytic cascades, inflammation, and ultimately resulting in neuronal death [17]. However, it was demonstrated that IHT is a protective approach and that IHT is used as an effective technique to improve human performance through adaptation to hypoxia. IHT can induce the expression of HIF-1, a critical dimeric protein involved in pathways responsible for hypoxia response [188]. HIF-1 increases vascularity in hypoxic regions, such as ischemic areas and tumors. This enhanced blood flow, in turn, improves neuronal viability. IHT can also induce the activation of anti-apoptotic kinases, including Akt and ERK [206]. IHT regulates the M2 polarization of activated microglia, lowers ROS production, and increases phagocytic activity. Furthermore, IHT increases the production of anti-inflammatory cytokines, such as IL-10 and IL-4 [17].

In a recent animal study, remote ischemic limb conditioning (RLC) polarized peripheral monocytes to a CCR2^+^ pro-inflammatory subset both in vitro and in vivo after MCAO. RLC caused a shift toward Lys6C^high^ in the microglia of the stroked brain. Animals subjected to RLC also had smaller infarct volumes, reduced brain swelling, and improved neuroglial functions [207]. A possible explanation is that the pro-inflammatory Lys6C^high^ microglia first infiltrate the brain in a CCR2-dependent pathway and then may differentiate into anti-inflammatory Lys6C^low^ microglia [208]. Supporting this idea, it was shown that selective inhibition of Lys6C^high^ microglia led to reduced M2 polarization, worse neurological recovery, and larger infarct volumes [209].

### Hypothermia

As a neuroprotective factor, hypothermia reduces the accumulation of post-stroke neuronal damage by reducing glutamate release and ROS production [18]. In an animal study by Ji et al., mild hypothermia attenuated the nuclear accumulations of several oxidative DNA lesions and reduced infarct volume [187]. Joseph et al. demonstrated that the expression of annexin A1, an important post-stroke pro-inflammatory protein, exhibited a reduction within polymorphonuclear cells (PMNs) located in the peri-lesional cortex after 48 h of hypothermia induced by exposure to hydrogen sulfide [210].

Treatment with mild hypothermia (33°C) 20- and 60-min post-MCAO led to a significant reduction in ACs and activated microglia and astrocytes, identified by active caspase-3, anti-CD-68, and glial fibrillary acidic protein (GFAP) immunohistochemistry. Ultimately, rats treated with mild hypothermia for 20 and 60 minutes post-stroke showed an improved neurological deficit score [211] and reduced infarct size [212].

## Applied molecular-directed strategies

Recent advancements in acute stroke therapy are encouraging, but further efforts are required to expand the reach and efficacy of reperfusion therapies, maximizing their potential benefit for a broader patient population [213]. Furthermore, aligning with expert recommendations, prioritizing the exploration of innovative therapeutic strategies can effectively inform the allocation of resources and research endeavors.

A promising area of research investigates the potential for combination therapies, employing both efferocytosis-directed and classical thrombolytic agents, to enhance recanalization efficacy. Recent studies have shown the utility of this approach in reducing the required thrombolytic dose and improving neurological outcomes. The ability of efferocytosis-directed agents to lengthen the time window of thrombolytics has not yet been assessed; however, their immunomodulatory effects make them a rationale candidate for future studies.

Recombinant tissue plasminogen activator (tPA) is now approved as the treatment of choice for early recanalization following an ischemic stroke [213]. tPA may act as a cytokine to induce migration of Iba-1^+^/CD68^+^ microglia, increase MMP-3, and disrupt the BBB. Interestingly, combination therapy of tPA with progesterone (PROG) attenuated these effects. Furthermore, tPA increased the expression of multiple M1 markers, such as iNOS, IL-1b, and TNF-a, while PROG+tPA suppressed M1 markers and upregulated M2 markers (IL-10 and Arg-1) [214]. Co-administration of annexin A2, a receptor for tPA and plasminogen, plus tPA, could enhance the efficacy of thrombolytic treatment. The combination therapy was able to reduce infarct volume, preserve BBB integrity, attenuate microglia activation, decrease the required tPA dose, and improve neurological outcomes, even better than tPA alone [215, 216].

Prophylactic intraperitoneal rosiglitazone (6 mg/kg) 1 h before and at the time of MCAO reduced the infarct volume, BBB disruption, and hemorrhagic transformation following tPA (10 mg/kg) administration. Moreover, by upregulating CD206 and Arg‐1 and downregulating iNOS, rosiglitazone polarized microglia toward the M2 phenotype. Higher counts of CD206^+^/Iba‐1^+^ and Arg‐1^+^/Iba‐1^+^ microglia were associated with less hemorrhagic area and BBB disruption, while iNOS^+^/Iba‐1^+^ microglia were positively correlated with them [217]. Supporting these findings, another study found that two weeks of prophylactic rosiglitazone combined with post-MCAO administration of tPA could reduce infarct volume and improve neurological outcome [218]. Rosiglitazone dampened vasculature damage by inhibiting MMP-9 activation, pro-inflammatory cytokine production, and preserving type IV basement membrane collagen. However, these beneficial effects were not achieved by the post-MCAO administration of rosiglitazone.

A recent study has uncovered a promising role for iron chelators in recanalization. Researchers showed that zinc (Zn^2+^), Fe^3+^, and Fe^2+^ inhibited the in vitro thrombolysis effects of tPA [219]. When a metal chelator, ethylenediaminetetraacetic acid (EDTA), was co-administered with tPA, it significantly facilitated tPA-induced thrombolysis. Furthermore, EDTA+tPA administration achieved higher rates of reperfusion in femoral artery occlusion. Along with iron chelators’ beneficial effects on ferroptosis, these results may indicate a potential capacity for metal chelators to improve efficacy and probably reduce required doses of tPA.

## Conclusion

In conclusion, efferocytosis is a crucial brain homeostatic process responsible for the removal of ACs and leads to neuroprotective and immunoregulatory effects in the insulted area. Defective efferocytosis can lead to secondary necrosis and inflammation and may be responsible for adverse outcomes after brain pathologies. Various potential therapeutic targets have been identified for enhancing efferocytosis, including OPN, CysLT2R, µ opioid receptor antagonist β-funaltrexamine, and mitochondria-targeted drugs (Table 1, Fig. 1). CD163 is an important efferocytosis-related biomarker of acute ischemic stroke and can help with stroke diagnosis and prognosis. Another important target for stroke treatment is ferroptosis. Numerous therapeutic agents, such as iron chelators, Fer-1, NAC, Se, CUR, and PPARγ and RXR agonists, have been investigated (Fig. 2). Several non-pharmacological approaches have also been studied, including physical rehabilitation, hypoxia/ischemia, and hypothermia. It is crucial to continue exploring these potential therapies to improve the outcomes of stroke and other brain pathologies. These strategies aim to restore homeostasis, reduce inflammation, and optimize the clearance of ACs. The identification of therapeutic targets and biomarkers related to efferocytosis provides a promising foundation for future research and the development of effective therapies for stroke recovery (Table 1). By harnessing the potential of efferocytosis, we can strive toward improving outcomes and enhancing the quality of life for stroke survivors. Further preclinical and clinical research is needed to fully understand the process of efferocytosis and its therapeutic potential in stroke and other neurological disorders.

**Table 1:** Summary of potential therapeutic targets and approaches for stroke recovery and their molecular basis.

Therapeutic Target/ Approach	Mechanism of Action	Refs
**Pharmacological treatments**		
OPN and its derivatives	M2 polarization, phagocytosis activation, reduction in local inflammation	[68–71]
PPARγ agonists (rosiglitazone, pioglitazone)	MAPKs and NF-κB pathway suppression, M2 polarization. Pioglitazone: synergism with Nrf2/ARE–GPX4 pathway and ferroptosis inhibition (ICH). Rosiglitazone: CD36 upregulation (ICH).	[72, 73, 144, 159]
Metformin	M2 polarization, increase in angiogenesis, and neurogenesis	[76]
CysLT2R selective antagonist (HAMI3379)	M2 polarization, NF-κB pathway suppression	[77]
miR-98	PAFR inhibition	[82]
IL-4	Microglial efferocytosis Enhancement	[81]
µ opioid receptor antagonist (β-funaltrexamine)	Suppression of NF-κB, CREB, TNF-α, IL-1β, NO, and PGE2, as well as M2 polarization and upregulation of Arg-1 and CD163	[78]
RXR agonist (bexarotene)	Axl and CD36 upregulation	[142, 143]
Fer1 and resveratrol	Inhibition of ROS production, SIRT1-UCP2 pathway activation	[146, 148]
Humanin	Upregulation of PPARγ and its target genes (e.g., MSD)	[186]
Mitochondria-targeted drugs	**NXY-059 & STAZN**: Free radical scavenger, reduction of neuronal loss	[178, 220, 221]
	**Resveratrol**: Inhibition of ROS production, SIRT1-UCP2 pathway activation	[179]
	**MRS2365 and 2meSADP**: astrocyte P2Y1 receptors activation	[180–182]
Fluoxetine	Inhibiting pro-inflammatory cytokine production and microglial oxidative stress, as well as facilitation of efferocytosis	[83]
Iron chelators (DFO, PIH, minocycline, LTF, DXZ, and DFP)	Reduction in intracellular iron load, cerebral edema, and ROS production (ICH)	[136–140]
GPX4 up-regulators (selenium, dauricine, baicalin, crocin, and curcumin)	Upregulating the expression of GPX4, inhibiting ferroptosis (ICH)	[153–158]
NAC	Inhibition of LOXs decreases in the toxic products of ARA (ICH)	[160]
IRN	Inhibiting ferroptosis, ROS production, and TP53, as well as upregulating miR-122-5p and SLC7A11 (ICH)	[161]
CDP-choline	Decrease in PC breakdown and FFA oxidation	[174, 175]
sCD163, microglia-bound CD163	Hemoglobin and iron sequestration, reducing neuroinflammation	[112–114]
**Non-pharmacological treatments**		
Physical rehabilitation	Activation of PI3K/Akt and SIRT1/FoxO1 pathways, increase in BDNF and Bcl-2, as well as suppression of caspase-3 and BAX	[189–197]
IHT	HIF-1 activation, increase in vascularity of hypoxic regions, anti-apoptotic kinases activation (e.g., ERK and Akt), M2 polarization, increase in anti-inflammatory cytokines (e.g., IL-10 and IL-4), and reduction in ROS production	[17, 188, 206]
Hypothermia	Reduction in glutamate release, ROS production, oxidative DNA lesions, and PMN annexin A1, as well as microglia and astrocytes activation	[18, 187, 210–212]

*AKT* Protein kinase B, *ARA* Arachidonic acid, *ARE* Antioxidant response element, *BDNF* Brain-derived neurotrophic factor, *CD* Cluster of differentiation, *CDP* Cytidine-5V-diphosphocholine, *CREB* Cyclic adenosine monophosphate (cAMP) response element-binding protein, *CysLT2R* Cysteinyl leukotriene 2 receptor, *DFO* Deferoxamine, *DFP* Deferiprone, *DXZ* Dexrazoxane, *ERK* Extracellular Signal-Regulated Kinase, *FFA* Free fatty acids, *FoxO* Forkhead box O, *GPX4* Glutathione peroxidase 4, *HIF-1* Hypoxia inducible factor-1, *ICH* Intracerebral Hemorrhage, *IHT* Intermittent hypoxia training; *IL* Interleukin, *IRN* Isorhynchophylline, *LOX* Lipoxygenase, *LTF* Lactotransferrin, *MAPKs* Mitogen-activated protein kinase, *MSD* Mitochondrial superoxide dismutase, *NAC* N–acetylcysteine, *NF-kB* Nuclear Factor Kappa B, *NO* Nitric oxide, *OPN* Osteopontin, *PAFR* Platelet activating factor receptor, *PC* Phosphatidylcholine, *PGE2* Prostaglandin E2, *PI3K* Phosphoinositide 3-kinase; *PIH* Pyridoxal Isonicotinoyl Hydrazone, *PMN* Polymorphonuclear cells, *PPAR* Peroxisome proliferator-activated receptor, *ROS* Reactive oxygen species; *RXR* Retinoid X Receptor, *sCD163* Soluble CD163, *SIRT1* Sirtuin 1, *UCP2* Uncoupling protein 2, *SLC7A11* Solute carrier family 7 member 11, *STAZN* Stilbazulenyl nitrone, *TNF-α* Tumor necrosis factor alpha, *TP53* Tumor protein p53.
